# The brief negative symptom scale: validation of the German translation and convergent validity with self-rated anhedonia and observer-rated apathy

**DOI:** 10.1186/s12888-016-1118-9

**Published:** 2016-11-22

**Authors:** Martin Bischof, Caitriona Obermann, Matthias N. Hartmann, Oliver M. Hager, Matthias Kirschner, Agne Kluge, Gregory P. Strauss, Stefan Kaiser

**Affiliations:** 1 Department of Psychiatry, Psychotherapy and Psychosomatics, Psychiatric Hospital, University of Zurich, Lenggstrasse 31, 8032 Zurich, Switzerland; 2 Laboratory for Social and Neural Systems Research, Department of Economics, University of Zurich, Bluemlisalpstrasse 10, 8006 Zurich, Switzerland; 3 Department of Psychology, State University of New York at Binghamton, Binghamton, NY 13902-6000 USA

**Keywords:** Schizophrenia, Negative symptoms, Rating scale, Assessment

## Abstract

**Background:**

Negative symptoms are considered core symptoms of schizophrenia. The Brief Negative Symptom Scale (BNSS) was developed to measure this symptomatic dimension according to a current consensus definition. The present study examined the psychometric properties of the German version of the BNSS. To expand former findings on convergent validity, we employed the Temporal Experience Pleasure Scale (TEPS), a hedonic self-report that distinguishes between consummatory and anticipatory pleasure. Additionally, we addressed convergent validity with observer-rated assessment of apathy with the Apathy Evaluation Scale (AES), which was completed by the patient’s primary nurse.

**Methods:**

Data were collected from 75 in- and outpatients from the Psychiatric Hospital, University Zurich diagnosed with either schizophrenia or schizoaffective disorder. We assessed convergent and discriminant validity, internal consistency and inter-rater reliability.

**Results:**

We largely replicated the findings of the original version showing good psychometric properties of the BNSS. In addition, the primary nurses evaluation correlated moderately with interview-based clinician rating. BNSS anhedonia items showed good convergent validity with the TEPS.

**Conclusions:**

Overall, the German BNSS shows good psychometric properties comparable to the original English version. Convergent validity extends beyond interview-based assessments of negative symptoms to self-rated anhedonia and observer-rated apathy.

## Background

The negative symptoms of schizophrenia consist of flattened affect, alogia, social withdrawal, avolition, and anhedonia [1]. They account to a large extent for impairments of work and social functioning, as well as longterm morbidity [2–4]. Today, pharmacological and psychosocial interventions have limited efficacy for reducing negative symptoms [5, 6].

The reliable and valid assessment of negative symptoms is critical for the development of pathophysiological models and effective treatments [7]. Since the introduction of quantitative assessment of negative symptoms in the early 1980s, concepts of negative symptoms have undergone subtle but important changes [8, 9]. Most importantly, this includes the distinction of two main factors of negative symptoms apathy/avolition and diminished expression [1, 10]. Other important shifts include the definition of cognitive dysfunction as a separate symptom dimension, the distinction between objective and subjective aspects of apathy/avolition, and the separation of consummatory and anticipatory anhedonia [9].

A consensus conference in 2005 under the auspices of the National Institute of Mental Health (NIMH) has provided an integration of these developments into a common conception of negative symptoms [9]. A main outcome of this consensus conference was the agreement that new instruments would have to be developed in order to fully cover this modern conceptualization of negative symptoms. Subsequently, two scales were developed – the Clinical Assessment Interview for Negative Symptoms (CAINS) [11] and the Brief Negative Symptom Scale (BNSS) [12]. The BNSS was explicitly intended for easy application in the context of clinical trials or experimental psychopathology studies. The BNSS is a 13-item instrument, which allows rapid assessment of negative symptoms based on a semi-structured interview. The authors were able to establish excellent psychometric properties in a series of validation studies [12–14]. Successful translations have been accomplished for Spanish and Italian versions of the BNSS, which also demonstrate excellent psychometric properties [15, 16].

The present study aimed to establish reliability and validity of the German version of the BNSS. In addition, we expanded previous investigations of convergent validity by discriminating between self-reported anticipatory and consummatory pleasure and by including observer-based measures of apathy/avolition. First, in order to assess whether the BNSS anhedonia subscales capture the self-rated subjective experience of anhedonia, we included the Temporal Experience of Pleasure Scale. Second, we assessed the relationship between BNSS ratings and observer-rated apathy/avolition by including the Apathy Evaluation Scale rated by the primary nurse. We also included the Modified Simpson-Angus Scale (MSAS) as a measure for extrapyramidal side effects in the estimation of divergent validity.

## Methods

### Participants

Seventy-five individuals meeting Diagnostic and Statistical Manual of Mental Disorders, Fourth Edition (DSM-IV) criteria for schizophrenia (*n* = 65) or schizoaffective disorder (*n* = 10, not currently in a mood episode) were included in the study. The local Ethics committee approved the study, and all participants gave written informed consent.

Inclusion criteria were: (1) outpatient or inpatient treatment at the Psychiatric Hospital, University of Zurich, (2) a clinical diagnosis schizophrenia or schizoaffective disorder and (3) age from 18 to 65 years. Importantly, all patients had to be clinically stable, i.e. they did not show positive symptoms of more than moderate severity (see exclusion criteria below) and received a constant dose of antipsychotic medication for at least two weeks prior to testing. Exclusion criteria were: (1) daily lorazepam dosage > 1 mg, (2) positive symptoms of moderate severity (Positive and Negative Syndrome Scale (PANSS) [17]; any positive subscale item score >4), (3) history of head injury or neurological disorder, (4) insufficient German fluency (5) additional DSM-IV axis-1 or axis-2 diagnostic criteria (according to the treating clinician). To confirm axis-1 diagnosis in patients and exclude comorbid axis-1 disorders, we used the Mini-International Neuropsychiatric Interview [18]. All patients were treated with second-generation antipsychotic medication.

Patients were either inpatients at the end of their hospitalization (*n = 49*) or outpatients (*n = 26*) treated at the Psychiatric Hospital, University of Zurich. Please note that the average inpatient stay for patients with schizophrenia in Swiss psychiatric hospitals is above 40 days [19]; as a consequence a lot of of our inpatients would be treated as outpatients in different healthcare systems. Notably, inpatients took part in a multimodal treatment program and were encouraged to participate in activities outside the hospital, which permitted assessment of apathy/avolition symptoms. The The treatment setting of our participants regularly includes a morning-meeting after breakfast, activation groups and various combinations of work-, occupational-, sports- and arts-therapies. These therapies take place from Monday to Friday during mornings and afternoons. Thus, the inpatient treatment program is comparable to a day-care setting. In addition to the group-based therapies, patients have individual sessions with a physician and/or a psychologist three times a week. Demographic information is presented in Table 1.

**Table 1:** Demographics (*n* = 75)

	Mean	(SD)
Age	31.5	10.9
Education (years)	11.6	2.9
Duration of illness (months)	133.2	224.1
Number of hospitalizations	4.7	3.9
% male	74.7	
% inpatients (vs outpatients)	65.3	
% Diagnosis Schizophrenia (vs Schizoaffective) Antipsychotic treatment	86.7	
% second generation	92.0	
% first and second generation	4.0	
% none	4.0	
PANSS total	50.8	12 .3
PANSS positive factor^a^	1.8	0.7
PANSS negative factor^a^	2.3	1.0
PANSS disorganized factor^a^	1.7	0.7
PANSS excited factor^a^	1.2	0.4
PANSS depressed factor^a^	1.7	0.8
CDSS total	1.8	2.2
GAF total	55.9	11.6

^a^mean = mean per item

### Procedures

The primary instrument of interest was the Brief Negative Symptom Scale (BNSS) [12]. As mentioned in the background section, the scale allows a rapid and reliable assessment of negative symptoms. It consists of 13 items and 6 subscales (anhedonia, distress, avolition, blunted affect and alogia, see Table 2) and can be split into the two different factors *motivation and pleasure* and *emotional expressivity* [14]. The scale includes a comprehensive manual, a workbook and a score sheet, which all have been translated into German. Negative symptoms were also assessed with the Scale for the Assessment of Negative Symptoms (SANS) [8], which is a widely used scale including 4 subscales (affective flattening/blunting, alogia, avolition/apathy and anhedonia/asociality).

**Table 2:** Descriptive statistics, item-total correlations, ICC’s

	M	SD	Skew	Kurtosis	Item-total score r	ICC^b^
Anhedonia						
1. Intensity of pleasure during activities	2.3	1.6	0.1	-0.9	0.80**	
2. Frequency of pleasurable activities	2.5	1.6	0.0	-0.8	0.78**	
3. Intensity of future pleasure	2.1	1.5	0.4	-0.3	0.69**	0.88
Subscale total	7.1	4.4	0.1	-0.6		
Distress subscale						
4. Lack of normal distress	1.0	1.4	1.3^a^	0.7	0.51**	0.93
Asociality subscale						
5. Asociality behavior	2.6	1.4	0.1	-0.3	0.70**	
6. Asociality inner-experience	2.0	1.4	0.6	-0.3	0.71**	
Subscale total	4.6	2.6	0.4	-0.3		0.95
Avolition subscale						
7. Avolition behavior	2.5	1.3	0.0	-0.6	0.74**	
8. Avolition inner-experience	2.1	1.5	0.2	-0.7	0.79**	
Subscale total	4.7	2.7	0.2	-0.5		0.87
Blunted affect subscale						
9. Facial expression	2.2	1.6	0.6	-0.4	0.85**	
10. Vocal expression	2.0	1.7	0.6	-0.5	0.81**	
11. Expressive gestures	2.1	1.7	0.4	-1.0	0.78**	
Subscale total	6.3	4.7	0.6	-0.5		0.95
Alogia subscale						
12. Quantity of speech	1.3	1.5	1.1^a^	0.9	0.72**	
13. Spontaneous elaboration	1.5	1.7	0.9	0.0	0.75**	
Subscale total	2.7	3.1	1.0	0.1		0.97
BNSS total score	26.3	14.7	0.7	0.1		0.97

^a^Skew towards nonpathological ratings

^b^ *n* = 28, Two-way mixed model, absolute agreement definition, average measures

**p < 0.01

The Global Assessment of Functioning scale (GAF) [20] and the Personal and Social Performance Scale (PSPS) [21] both measure functioning. However, the latter excludes psychopathological aspects and is therefore a more specific instrument for functioning. The Calgary Depression Scale for Schizophrenia (CDSS) [22] is an interview-based instrument that has been developed to identify depressive symptoms in patients with schizophrenia. The Positive and Negative Syndrome Scale (PANSS) [17] is a 30-item scale that is split into three dimensions (negative symptoms, positive symptoms and general psychopathology including depression and anxiety) and allows to capture a broad range of symptomatology. However, based on recent findings about the underlying structure of the PANSS, a 5-factor model (positive, negative, disorganized/concrete, excited and depressed) described by Wallwork et al. was used for all calculations [23].

The five clinical raters, who completed ratings for all scales, were trained and regularly supervised by the senior author. Raters were trained individually, which included a step-by-step instruction on how to use the scale, joint ratings of videos and live interviews and conduction of supervised interviews. To increase interrater reliability, raters were additionally trained in video-based group sessions. Three of them have a masters-degree in clinical psychology and two were psychiatrists in training.

The German version of the BNSS is based on a translation process including forward translation, back translation, and reconciliation of back translation with the BNSS developers. This process therefore met the highest industry standards. To extend the evaluation of convergent/discriminant validity beyond the original English BNSS studies, the Apathy Evaluation Scale (AES-C) [24] was administered by the primary nurse for the inpatients only. The AES-C was established to measure apathy for neurological and psychiatric patients. The Temporal Experience of Pleasure Scale (TEPS) [25] was filled out by the patients for anhedonia self-report. It allows to distinguish consummatory and anticipatory anhedonia which is specifically important in the role of schizophrenia. Finally, the Modified Simpson-Angus Scale (MSAS) [26] was completed by the physician raters to evaluate extrapyramidal symptoms.

To assess inter-rater reliability, two different assessors rated 27 patients independently within the same session. The raters attended the same interview in person but were then separated to complete ratings.

### Statistical analysis

To calculate correlations for convergent and discriminant validity, Pearson’s r was computed. A partial correlation between negative symptoms (BNSS total) and functioning (GAF) was conducted in order to control the influence of common sources of secondary negative symptoms on this association. Reliability was calculated with Cronbach’s alpha for internal consistency and Intraclass Correlation Coefficient (ICC) for inter-rater agreement. Statistical analyses were performed using IBM SPSS Statistics Version 21. A test for significant differences in the magnitude of the correlation between the BNSS and the TEPS pleasure scales was performed using Steiger’s method implemented in the online tool http://quantpsy.org/corrtest/corrtest2.htm [27, 28].

## Results

### Descriptive statistics and distribution of scores

Descriptive statistics for BNSS items and subscales are presented in Table 2. The lack of normal distress as well as the quantity of speech items had skew > 1.0 indicating distribution towards nonpathological ratings.

### Internal consistency

Cronbach’s alpha, computed to analyze internal consistency, was 0.93, indicating that the items measure a distinct latent construct of negative symptoms. Item total correlations showed that all BNSS items were significantly correlated with the BNSS total scale score (Table 2). Alpha if-item-deleted coefficients ranged from 0.92 to 0.93, with only the lack of normal distress item lowering the value.

### Inter-rater reliability

To assess inter-rater reliability (Table 2), Intraclass Correlation Coefficients (ICCs) were calculated for the BNSS total score and for each subscale. The ICC for the BNSS total score was 0.97, and ICC values for the subscales were: Anhedonia 0.88, distress 0.93, asociality 0.95, avolition 0.87, blunted affect 0.95, and alogia 0.97.

### Convergent validity with clinician-rated negative symptoms and functioning

To assess convergent validity (Table 3) we used clinician rated scales (SANS, PANSS). The BNSS total was strongly correlated with the SANS Total (*r* = 0.89, *p* < 0.001) and PANSS Negative factor (i.e., emotional withdrawal, poor rapport, passive/apathetic social withdrawal, lack of spontaneity and motor retardation) (*r* = 0.89, *p* < 0.001), suggesting good convergent validity with other negative symptom measures.

**Table 3:** Convergent validity

	BNSS total	BNSS motivation/pleasure	BNSS diminished expression	BNSS anhedonia
PANSS negative factor	0.89**	0.71**	0.90**	
SANS avolition/apathy	0.63**	0.65**	0.47**	0.43**
SANS asociality/anhedonia	0.77**	0.80**	0.57**	0.58**
SANS blunted affect	0,82**	0.56**	0.91**	
SANS alogia	0.63**	0.37**	0.77**	
SANS total	0.89**	0.73**	0.87**	
AES total (*n* = 48)	-0.48**	-0.50**	-0.40**	
GAF	-0.69**	-0.68**	-0.56**	
PSP total	-0.73**	-0.73**	-0.57**	
	TEPS anticipatory	TEPS consummatory		
BNSS intensity of pleasure	-0.25*	-0.29*		
BNSS intensity of future pleasure	-0.26*	-0.20		
BNSS anhedonia subscale	-0.31*	-0.36*		
SANS asociality/anhedonia	-0.13	-0.15		

Note. *p < 0.05; **p < 0.01

Good subscale-level convergent validity was indicated by moderate to high correlations between BNSS subscale scores and the average of items comprising the 4 subscales of the SANS: BNSS anhedonia with SANS anhedonia/asociality (*r* = 0.58, *p* < 0.001); BNSS asociality with SANS anhedonia/asociality (*r* = 0.79, *p* < 0.001); BNSS avolition with SANS avolition (*r* = 0.79, *p* < 0.001); BNSS blunted Affect with SANS blunted Affect (*r* = 0.93, *p* < 0.001); and BNSS alogia with SANS alogia (*r* = 0.82, *p* < 0.001).

The BNSS lack of normal distress item was negatively correlated with the PANSS depressed factor (*r* = −0.33, *p* < 0.001), supporting the item’s validity.

The BNSS total score had a high inverse correlation with the GAF total (*r* = -0.69, *p* < 0.001) score, as well as the PSP total score (*r* = -0.70, *p* < 0.001), suggesting good convergent validity with functional outcome. A partial correlation was conducted, controlling for depression, anxiety, psychosis and disorganization, to determine whether this association remained significant when accounting for common sources of secondary negative symptoms. The correlation between the BNSS total and the GAF total remained highly significant (*r* = -0.62, *p* < 0.001).

### Convergent validity with self-rated anhedonia and observer-rated apathy

The TEPS self-report scale (Table 3) was used for convergent validity of the anhedonia subscale. The self-report scale was filled out by a smaller subsample (*n* = 66). The BNSS anhedonia subscale was negatively correlated with both the anticipatory (*r* = −0.31, *p* < 0.05) and consummatory (*r* = −0.36, *p* < 0.001) pleasure scales from the TEPS. Regarding the attempt to discriminate between anticipatory and consummatory pleasure, the BNSS anticipatory (*r* = -0.26, *p* < 0.05) as well as the BNSS intensity of pleasure item (*r* = -0.25, *p* < 0.05) were correlated with the TEPS anticipatory pleasure scale. On the other hand, only the BNSS consummatory (*r* = -0.29, *p* < 0.05) but not the anticipatory item (*r* = -0.20, *p* > 0.05) was correlated with the TEPS consummatory pleasure scale. However, the two correlation coefficients were not significantly different. The SANS anhedonia–asociality scale was neither correlated with TEPS anticipatory pleasure scale (*r* = −0.13, *p* > 0.05) nor the consummatory pleasure scale (*r* = -0.15, *p* > 0.05). These results support the convergent validity of the BNSS anhedonia items.

The AES observer scale (Table 3) was completed by the inpatient’s primary nurse (*n* = 49) and correlated moderately with the BNSS total score (*r* = 0.48, *p* < 0.001) and the BNSS motivation/pleasure scale (i.e., average of anhedonia, avolition, asociality items) (*r* = 0.50, *p* < 0.001). Thus, BNSS ratings correspond well to apathy ratings by the primary care person.

### Discriminant validity

Discriminant validity (Table 4) of the BNSS was examined by calculating correlations with positive symptoms, disorganized symptoms, general symptoms, and extrapyramidal side effects (MSAS). BNSS total was not significantly correlated with the PANSS positive factor (*r* = -0.02, *p* > 0.05), PANSS depressed factor (*r* = 0.02, *p* > 0.05) or PANSS excited factor (*r* = 0.19, *p* > 0.05). There was a modest correlation between the PANSS disorganized factor and the BNSS total score (*r* = 0.37, *p* < 0.001). This correlation was mainly due to the Expression subscales (range *r* = 0.40–0.42, *p* < 0.01), whereas no correlation with the subscales of the Motivation and Pleasure factor was observed (range *r* = -0.04 – 0.23, *p* > 0.05). However, the mean score on the disorganization factor was 5.00 (2.12) (lowest possible 3) and the maximum score 10 (from 21 possible), therefore representing a non-disorganized sample. The CDSS total score was not significantly correlated with the BNSS total score (*r* = 0.16, *p* > 0.05) or the BNSS anhedonia score (*r* = 0.08, *p* > 0.05). Finally, the BNSS total score was not correlated with the MSAS (*r* = 0.12, *p* > 0.05).

**Table 4:** Discriminant validity

	PANSS positive	PANSS depressed	PANSS disorganized	PANSS excited	CDSS	MSAS^a^
BNSS total	-0.02	0.02	0.37**	0.19	0.16	0.12
SANS total	0.07	0.14	0.42**	0.29*	0.22	0.10
PANSS negative	-0.01	0.16	0.41**	0.30**	0.26	0.15
BNSS anhedonia	-0.13	-0.05	0.23	0.07	0.08	0.21

^a^ *n* = 56

*p < 0.05; **p < 0.01

## Discussion

Overall the German translation of the BNSS shows good psychometric properties including inter-rater reliability, which mostly replicate the findings for the original version. Importantly, we show that overall the BNSS anhedonia subscales converge with self-reported anhedonia, although the differentiation between anticipatory and consummatory anhedonia remains a challenge. In addition, BNSS rated negative symptoms correlate with observer-rated apathy as assessed by the primary nurse.

Convergent validity with the SANS total and the PANSS negative factor was high and moderate to high for the respective subscales. It has to be noted that the observed correlations might be somewhat higher than expected, because the same rater scored the BNSS and the other negative symptom scales. Nevertheless, these results confirm the assumption of a common and established construct for negative symptoms. We also found strong associations between negative symptoms and functioning, emphasizing the importance of its role as a predictor of poor outcome [2].

For anhedonia, we could show incremental validity as the TEPS scales only correlated with the BNSS anhedonia scale, but not with the SANS anhedonia subscale. This strongly suggests that BNSS anhedonia captures the subjective experience of anhedonia better than older negative symptom scales. The importance of the distinction between anticipatory and consummatory pleasure has been emphasized, as only the former has been consistently found to be impaired in patients with schizophrenia [25]. We also addressed the question of whether the individual BNSS anhedonia items show specific correlations with the respective TEPS anticipatory and consummatory subscales. However, this distinction was limited, as all correlation coefficients were in a similar range. Only the BNSS intensity of pleasure item seemed to be somewhat more strongly related to the TEPS consummatory anhedonia subscale, but differences between correlations were small and non-significant. The lack of convergent validity between the TEPS subscales and BNSS anhedonia items could reflect limitations of the BNSS, TEPS, or both measures in evaluating consummatory and anticipatory pleasure. The TEPS consummatory and anticipatory pleasure subscales have shown somewhat diverging findings across studies, with some investigations showing a deficit in consummatory but not anticipatory [29] and others an impairment in anticipatory but not consummatory pleasure [25]. This raises the question whether the TEPS was fully suited for assessing the specific convergent validity of the items with the anhedonia subscale. Also, the BNSS intensity item may show restricted range and rely on memory aspects that might be impaired in patients, suggesting that few patients may have a true consummatory pleasure deficit [30]. Thus, it remains an open question whether the current convergent validity findings on anhedonia reflect limitations of the scales or the conceptualization of anhedonia as a consummatory vs. anticipatory pleasure deficit in schizophrenia.

To expand prior work on convergent validity to a different assessment modality, we included the AES as an observer rating of apathy that was completed by the patient’s primary nurse. We found moderate correlations between AES ratings and BNSS total and motivation/pleasure scores. This is quite remarkable, because the nurses were not trained in the assessment of negative symptoms and were simply instructed to score the AES based on their observations in daily life on the ward. Interview-based assessments always raise the question whether the participant correctly reports his interests and activities [31], but the present findings suggest a good convergence between interview-based reports and observer ratings. Whether this convergence generalizes to non-professional care-givers such as relatives remains an open issue.

Discriminant validity was shown by excellent differentiation between the BNSS and measures of positive symptoms and depression. It can therefore be concluded that the BNSS clearly measures disturbances that are different from other symptom dimensions of schizophrenia. In addition, extrapyramidal side effects have been considered as major cause for secondary negative symptoms. The only study with the BNSS to have previously included a measurement of extrapyramidal symptoms has taken a categorical approach of present or absent extrapyramidal side effects [16]. Here, we have employed a dimensional approach, which shows that BNSS total and subscales were not associated with extrapyramidal symptoms. This is in line with the categorical findings by Mucci and colleagues, thereby supporting discriminant validity.

Some limitations of the present study need to be considered. First, overall the participants showed relatively low levels of positive, depressive, and extrapyramidal symptoms. Therefore, it is an important question whether the excellent discriminant validity reported here generalizes to populations with higher non-negative symptom levels. Third, the inclusion criteria may impact the ability of these findings to generalize to samples with greater stability and comorbid substance use disorders. Finally, the interpretation of convergent validity analyses is complicated by the use of different different forms of assessment (i.e., self-report questionnaires vs. clinician rated) and different types of raters (e.g., nurse vs. clinician). As such, method and rater variance should be accounted for when interpreting the correlations between BNSS scores and measures of anhedonia and apathy.

## Conclusions

The outcome of this study suggest that the BNSS can be used to assess negative symptoms across different languages and health care systems. Furthermore, our data show that convergent validity extends to self-ratings of anhedonia and observer-ratings of apathy. Overall, the BNSS is a promising instrument for the investigation of pathophysiological mechanisms underlying the negative symptoms of schizophrenia and particularly the two dimensions apathy/avolition and diminished expression. Most importantly, it allows assessment of negative symptoms across cultures and thus could possibly be of use in future international multicenter trials that target negative symptoms.

## Abbreviations

AES: Apathy evaluation scale
BNSS: Brief negative symptom scale
CDSS: Calgary depression scale for Schizophrenia
CAINS: Clinical assessment interview for negative symptoms
DSM-IV: Diagnostic and statistical manual of mental disorders, fourth edition
GAF: Global assessment of functioning scale
ICC: Intraclass correlation coefficient
MSAS: Modified simpson-angus scale
PANSS: Positive and negative syndrome scale
PSPS: Personal and social performance scale
SANS: Scale for the assessment of negative symptoms
TEPS: Temporal experience pleasure scale
